# Autologous Hematopoietic Stem Cell Transplantation for Autoimmune Diseases: From Mechanistic Insights to Biomarkers

**DOI:** 10.3389/fimmu.2018.02602

**Published:** 2018-11-16

**Authors:** Kelen Cristina Ribeiro Malmegrim, João Rodrigues Lima-Júnior, Lucas Coelho Marlière Arruda, Júlia Teixeira Cottas de Azevedo, Gislane Lelis Vilela de Oliveira, Maria Carolina Oliveira

**Affiliations:** ^1^Department of Clinical Analysis, Toxicology and Food Science, School of Pharmaceutical Sciences of Ribeirão Preto, University of São Paulo, Ribeirão Preto, Brazil; ^2^Center for Cell-based Therapy, Regional Hemotherapy Center of Ribeirão Preto Medical School, University of São Paulo, Ribeirão Preto, Brazil; ^3^Biosciences Applied to Pharmacy Program, School of Pharmaceutical Sciences of Ribeirão Preto, University of São Paulo, Ribeirão Preto, Brazil; ^4^Division of Rheumatology, Allergy, Immunology and Immunotherapy, Department of Internal Medicine, Ribeirão Preto Medical School, University of São Paulo, Ribeirão Preto, Brazil; ^5^Department of Clinical Science, Intervention and Technology, Karolinska Institutet, Stockholm, Sweden; ^6^Basic and Applied Immunology Program, Ribeirão Preto Medical School, University of São Paulo, Ribeirão Preto, Brazil; ^7^São Paulo State University (UNESP), Institute of Biosciences, Humanities and Exact Sciences (IBILCE), São Jose do Rio Preto, São Paulo, Brazil

**Keywords:** hematopoietic stem cell transplantation, immune reconstitution, autoimmune diseases, biomarkers, immune tolerance, immunoregulation

## Abstract

Phase I/II clinical trials of autologous hematopoietic stem cell transplantation (AHSCT) have led to increased safety and efficacy of this therapy for severe and refractory autoimmune diseases (AD). Recent phase III randomized studies have demonstrated that AHSCT induces long-term disease remission in most patients without any further immunosuppression, with superior efficacy when compared to conventional treatments. Immune monitoring studies have revealed the regeneration of a self-tolerant T and B cell repertoire, enhancement of immune regulatory mechanisms, and changes toward an anti-inflammatory milieu in patients that are responsive to AHSCT. However, some patients reactivate the disease after transplantation due to reasons not yet completely understood. This scenario emphasizes that additional specific immunological interventions are still required to improve or sustain therapeutic efficacy of AHSCT in patients with AD. Here, we critically review the current knowledge about the operating immune mechanisms or established mechanistic biomarkers of AHSCT for AD. In addition, we suggest recommendations for future immune monitoring studies and biobanking to allow discovery and development of biomarkers. In our view, AHSCT for AD has entered a new era and researchers of this field should work to identify robust predictive, prognostic, treatment-response biomarkers and to establish new guidelines for immune monitoring studies and combined therapeutic interventions to further improve the AHSCT protocols and their therapeutic efficacy.

## Introduction

More than 20 years ago, autologous hematopoietic stem cell transplantation (AHSCT) was proposed as an alternative and innovative treatment for severe and refractory autoimmune diseases (AD) (1, 2). This therapeutic approach has been successfully used to treat several AD and over the years phase I/II clinical studies have led to increased safety and efficacy of the procedure (1, 2). More recently, phase III randomized trials have proven greater therapeutic efficacy of AHSCT than conventional therapies for some AD, such as multiple and systemic sclerosis. In addition, these trials have demonstrated that AHSCT can induce long-term disease remission without further use of immunosuppression (3–11).

Despite the overall positive outcomes of the procedure, subgroups of patients fail to remain in remission after AHSCT and reactivate the autoimmune disease (1, 2). The factors associated with disease reactivation, however, remain to be investigated. They may range from patient-specific (e.g., disease physiopathology, age, co-morbidities) to more general factors (e.g., infections, resurgence of autoreactive T, and B cells) (1, 12).

In the last decade, immune monitoring analyses have shown that AHSCT is able to regenerate a new auto-tolerant immune T and B cell repertoire, increase immune regulatory mechanisms, and induce changes to an anti-inflammatory milieu in patients with different AD (13–44). Most of the established immune mechanisms of AHSCT are common to AD in general, while others may be disease-specific (1, 2, 12). Few studies have analyzed the mechanistic results by comparing data of autoimmune patients with different outcomes after AHSCT (17, 18, 26, 31). Table 1 lists all 39 mechanistic studies of AHSCT for AD already published since 2004 and the most common immune mechanisms reported.

**Table 1:** Described immune mechanisms of AHSCT in patients with autoimmune diseases.

**Disease**	**References**	**Immune mechanism**								

		**Renewal of the TCR Repertoire**	**Reactivation of thymic function**	**Modulation of gene expression**	**Regulatory** **T expansion**	**Regulatory B expansion**	**Homeostatic proliferation/** **CD8**^+^**CD28**^−^**CD57**^−^**expansion**	**Increased** **PD-1 expression on T and B cells**	**Changes in cytokine patterns**	**Decreased autoreactivity early post-AHSCT**
Multiple Sclerosis	Sun et al. (13)									
	Muraro et al. (14)									
	Dubinsky et al. (25)									
	Darlington et al. (36)									
	Abrahamsson et al. (39)									
	Muraro et al. (40)									
	de Paula Sousa et al. (41)									
	Arruda et al. (42)									
	Arruda et al. (45)									
	Oliveira et al. (44)									
	Karnell et al. (15)									
	Cull et al. (46)									
	Darlington et al. (36)									
Type 1 Diabetes	Li et al. (16)									
	Ye et al. (38)									
	de Oliveira et al. (17)									
	Zhang et el. (47)									
	Malmegrim et al. (18)									
Systemic Sclerosis	Farge et al. (19)									
	Bohgaki et al. (20)									
	Tsukamoto (21)									
	Baraut et al. (22)									
	Farge et al. (7)									
	Arruda et al. (48)									
	Arruda et al. (23)									
Systemic Lupus Erythematosus	Alexander et al. (24)									
	Zhang et al. (26)									
	Wada et al. (27)									
	Alexander et al. (28)									
Crohn's Disease	Burt et al. (29)									
	Clerici et al. (30)									
	Le Bourhis et al. (49)									
Juvenile Idiopathic Arthritis	de Kleer et al. (31)									
	Brinkman et al. (32)^*^									
	Wu et al. (33)									
	Delemarre et al. (34)									
Juvenile Dermatomyositis	Szodoray et al. (35)									
	Enders et al. (37)									
Other Rheumatic Diseases	Szodoray et al. (35)									
	Váróczy et al. (50)^*^									

* *These studies included only peripheral blood immunophenotyping analyses*.

**Figure 1:**
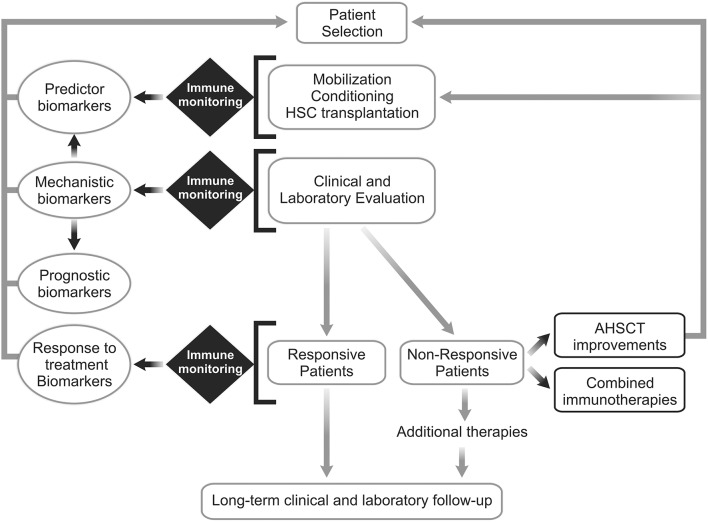
Future directions for establishment of biomarkers of autologous hematopoietic stem cell transplantation for autoimmune diseases. Patients with autoimmune disease (AD) must be are selected to undergo autologous hematopoietic stem cell transplantation (AHSCT). Based on the clinical, laboratory and immune monitoring results, patients will be classified in “responsive” or “non-responsive” to AHSCT. Well-performed immune monitoring evaluations are essential to settle mechanistic biomarkers and reveal new prognostic, predictor and/or response to treatment biomarkers. Further, additional therapeutic interventions can be proposed to treat patients who do not respond sufficiently to transplantation as a single treatment or reactivate the disease after some period of remission. New approaches for improving AHSCT efficacy in AD patients and/or combined immunotherapies are warranted.

In this context, understanding of the immune mechanisms that lead to different clinical outcomes is essential to refine AHSCT protocols or to propose adjuvant/combined immunotherapy. Figure 1 indicates future directions to establish biomarkers of AHSCT for AD, including sample biobanking of multicenter randomized clinical studies and development of adjuvant/combined immunotherapy for patients that did not respond to AHSCT as single therapy.

In this perspective, we discuss the current knowledge about the immune mechanisms involved in AHSCT for AD, suggest recommendations for further immune monitoring studies and propose future directions in the field.

## Immune mechanisms after AHSCT for AD: current status

The rationale of AHSCT is the eradication of the autoreactive immunological memory and regeneration of the immune system. Ablation of the immune system, including depletion of autoreactive memory T and B cells, is followed by the reestablishment of immune tolerance (51). However, exactly how AHSCT corrects a deregulated immune system is not yet completely understood (1, 2).

Since 2004, and over the past 14 years, several immune mechanisms after AHSCT for AD have been described (Table 1). Many groups have investigated how the immunological renewal after AHSCT for ADs may reset a deregulated immune system into a self-tolerant status, inducing long-term remission (1, 2). Patients have been prospectively evaluated in studies that elucidate some immune mechanisms (Table 1). Currently, we may consider that these results, already reproduced on different patient cohorts, diseases, conditioning regimens, and laboratories around the world, are quite robust and consistent. Nevertheless, we still need to extend and worldwide standardize the immune monitoring evaluations in AD patients to enable the discovery of biomarkers, which will ultimately help to improve AHSCT protocols and their therapeutic efficacy (Table 2).

**Table 2:** Requirements for immune monitoring analyses in patients with AD undergoing AHSCT.

**BIOLOGICAL SAMPLE**	**TIME-POINTS OF ANALYSES**	**RECOMMENDED ANALYSES**	**METHODS**
**Minimum requirements for immune monitoring, biobanking and biomarker identification**			
Serum/Plasma	At baseline (before mobilization) and at 1, 3, 6, 9, 12, 18, 24, 30, 36 months after AHSCT and annually thereafter Serum and plasma samples storage at−80°C	Total immunoglobulin levels (IgG, IgA, IgM)	ELISA
		Soluble biomarkers (TNF-α, IFN-γ, IL-2, IL-4, IL-6, IL-8, IL-17, IL-18, IL-10, TGF-β)	ELISA, multiplex
Total peripheral blood or PBMCs	At baseline (before mobilization) and at 1, 3, 6, 9, 12, 18, 24, 30, 36 months after AHSCT and annually thereafter PBMC samples cryopreservation and storage at N_2_ liquid for future functional assays	Blood cell counts (essential to calculate absolute numbers of immune cell subsets)	Hematology Analyzer
		Immunophenotyping of T, B, NK cell subsets (see Table 3) on fresh blood samples	Flow Cytometry, CyTOF (mass cytometry)
DNA (from PBMC)	At baseline (before mobilization) and at 3, 6, 9, 12, 24, 30, 36 months after AHSCT and annually thereafter DNA samples storage at−20°C	TREC and KREC levels	Multiplex real-time PCR
RNA (from PBMC)	At baseline (before mobilization) and at 6, 12, 18, 24 months after AHSCT and annually thereafter cDNA samples storage at −20°C	–	–
**Additional recommendations for immune monitoring and biomarker discovery**			
GrafT cells	At graft collection	Immunophenotyping of T, B, NK cell subsets (see Table 3) on fresh samples	Flow Cytometry, CyTOF (mass cytometry)
RNA (from PBMC)	At baseline (before mobilization) and at 6, 12, 18, 24 months after AHSCT and annually thereafter	B cell receptor (BCR) and/or T cell receptor (TCR) repertoire	NGS
		Gene expression, MicroRNA expression	Microarrays, PCR arrays, Real-time PCR
PBMCs or sorted cell subset	At baseline and at 1, 3, 6, 9, 12, 18, 24 months after AHSCT and annually thereafter Protein, DNA and/or RNA extraction	Proteomics Genomics (genome-wide association studies of polymorphisms) and epigenomics (epigenetic modifications) Transcriptomics (transcriptional signatures of tissues, cell population or single-cell)	Mass spectrometry, protein or peptide microarrays, aptamers High-Throughput DNA sequencing RNA sequencing, Microarrays
**Disease-specific recommendations for immune monitoring and biomarker discovery**			
Serum/plasma	At baseline (before mobilization) and at 1, 3, 6, 9, 12, 18, 24, 30, 36 months after AHSCT and annually thereafter	Specific autoantibody titers	ELISA
		Complement component levels	ELISA
		Specific disease surrogate soluble biomarkers	ELISA, multiplex
		Proteomics of extracellular microvesicles	Mass spectrometry
Total peripheral Blood (in EDTA) or PBMCs	At baseline (before mobilization) and at 1, 3, 6, 9, 12, 18, 24, 30, 36 months after AHSCT and annually thereafter PBMC samples cryopreservation at N_2_ liquid for future functional assays	Immunophenotyping of specific cell subsets (such as innate lymphoid cells; gut-homing T cells; skin-homing T cells; specific cell subset already demonstrated as surrogate/mechanistic biomarkers) Expression of PD-1, Lag-3, Tim-3, and TIGIT (co-inhibitory receptors with specialized functions in immune regulation) on T cells	Flow Cytometry, CyTOF (mass cytometry)
		Autoantigen-specific T cells (autoreactive cells)	Tetramer staining by flow cytometry
PBMCs or sorted cell subset	At baseline (before mobilization) and at 1, 3, 6, 9, 12, 18, 24 months after AHSCT and annually thereafter Protein, DNA and/or RNA extraction	Proteomics Genomics (genome-wide association studies of polymorphisms) and epigenomics (epigenetic modifications) Transcriptomics (transcriptional signatures of tissues, cell population or single-cell)	Mass spectrometry, protein or peptide microarrays, aptamers High-throughput DNA sequencing RNA sequencing, Microarrays
RNA from PBMC	At baseline (before mobilization) and at 6, 12, 18, 24 months after AHSCT and annually thereafter	MicroRNA expression	PCR arrays, Real-time PCR
Tissue biopsies (e.g., gut, skin)	At baseline (before mobilization) and at 6, 12, 18, 24 months after AHSCT and annually thereafter Protein and RNA extraction	Protein expression Gene expression	Immunofluorescence, Immunohistochemistry PCR arrays, Real-time PCR
Other biological fluid (e.g., cerebrospinal fluid)	At baseline (before mobilization) and at 6, 12, 18, 24 months after AHSCT and annually thereafter	Oligoclonal bands	Isoelectric focusing, followed by immunoblotting
		Immunophenotyping of specific cell subsets	Flow Cytometry, CyTOF (mass cytometry)

*PBMC, Peripheral Blood Mononuclear Cells; NGS, Next Generation Sequencing; TREC, T-cell receptor excision circles; KREC, κ-deleting recombination excision circles*.

### Common immune mechanisms after AHSCT for AD

#### Reactivation of thymus function and renewal of the TCR repertoire

Thymus function translates into production of naive T cell populations and their exportation to the periphery (52). Generation of a diverse T cell repertoire throughout life is essential for immunity against pathogens. During development, T cells undergo central tolerance mechanisms, where positive selection ensures that T cells recognize self-MHC molecules and negative selection eliminates most T cells specific to autoantigens (52–54).

Thymic production of early naive T cells, named recent-thymic emigrants (RTE), can be determined through analysis of signal-joint T cell receptor excision circles (sjTRECs) (51). TRECs are DNA byproducts of T cell receptor (TCR) gene rearrangements that take place during T cell development in the thymus, and are reliable markers of RTE production and thymic function (55).

In the context of AHSCT, thymic rebound, which is defined by volumetric enlargement and functional reactivation of the thymus following lymphoid ablation, has been reported (14, 32, 55). Lymphoid and myeloid cells may be partially or completely depleted, depending on the intensity of the AHSCT conditioning regimen. Shortly after AHSCT, immune reconstitution predominantly relies on peripheral expansion of T and B cells that survived the highly immunosuppressive regimen or that were re-infused with the stem cell graft (15, 56).

Muraro et al. (14) were the first to demonstrate the so-called “immune resetting” mechanism in multiple sclerosis (MS) patients treated with AHSCT. Increased TREC levels indicated that peripheral T cells underwent thymic maturation after transplantation (14, 51). In addition, deep sequencing analysis demonstrated extensive replacement of a pre-existing TCR repertoire with new T cell clones that have emerged after transplantation. In fact, greater TCR repertoire diversity was found in patients with complete clinical response, indicating an interesting clinical correlation (18). In the following years, other researchers have also demonstrated, in different AD, that T cells could regenerate in adults with inactive thymuses (13–15, 18, 27, 31, 34, 35, 40, 41) and that the TCR repertoire after AHSCT was indeed renewed (13, 14, 16, 18, 26–28, 31, 34) (Table 1).

In summary, thymus reactivation and renewal of the TCR repertoire following AHSCT are the most significant mechanisms of action of this therapy so far described. Therefore, we should strongly recommend routine analyses of TREC levels in patients with AD undergoing AHSCT (Table 2). In addition, whenever possible, TCR repertoire evaluation by next generation sequencing should also be performed (Table 2).

#### Modulation of gene expression

Transcriptional analyses are currently used to describe disease signatures, evaluate response to treatments and to define patient subgroups, among other applications. However, to date few studies have evaluated modulation of gene expression of immune cells in the context of AHSCT for AD. Our group has evaluated the gene and microRNA expression profiles of reconstituted immune cells after AHSCT in MS patients. de Paula Sousa et al. (41) analyzed the global gene expression profiling of peripheral CD4^+^ or CD8^+^ T cells from MS patients at pre-transplantation and periodically after AHSCT. Hierarchical gene clustering analysis revealed that at 2 years after AHSCT, CD8^+^ T cells from MS patients were more similar to samples from healthy controls (19).

Other studies demonstrated normalization of deregulated gene expression following AHSCT for multiple sclerosis (20, 22, 25). Arruda et al. (23) demonstrated post-transplant normalization of the expression of mir-16, mir-155 and mir-142-3P, which have immmunoregulatory functions and are abnormally expressed in MS patients (20). As expected, expressions of their putative target genes, FOXP3, FOXO1, PDCD1, and IRF2BP2, were increased at 2 years post-transplantation (20) (Table 1).

In transplanted type 1 diabetes patients, Ye et al. (38) observed increased IL10, TGFβ, and FOXP3 mRNA expression, despite no significant regulatory T cell expansion (44).

In conclusion, few studies have so far explored transcriptomic analyses to understand the immune mechanisms of AHSCT for AD. One important challenge is to perform global transcriptome or single gene expression analysis for specific genes in purified T and B cell subsets from patients that undergo AHSCT, before and after disease reactivation. Lack of proper study design for appropriate cell separation/sorting and RNA isolation and/or technology cost are the most probable reasons for researchers not having so far explored this approach. In our opinion, this is a powerful method that should be explored in future studies. Transcriptional signatures of diseases for diagnosis, mechanisms and response to treatment are currently needed. Besides transcriptomics, other advanced new tools and technologies may be also useful to identify therapeutic biomarkers, such as proteomics, cytomics, lipidomics, and metabolomics (57–61).

#### Changes in cytokine patterns

Many mechanistic studies have demonstrated that AHSCT decreases the inflammatory status of patients with different AD (13, 24, 28, 37, 38, 43, 51–53) (Table 1). Sun et al. (13) reported the first study that deeply evaluated the immune reconstitution in MS patients treated with AHSCT (13). Serum levels of IL-12, IFN-γ, TNF-α, IL-10, and IL-4 were modulated after transplantation, however they decreased to baseline levels at 12 months (13). The authors also reported significant transient increase of IFN-γ, TNF-α, and IL-10 serum levels between 3 and 6 months after AHSCT, compared to the pre-transplant period. On the other hand, IL-12 serum levels consistently decreased in all patients following AHSCT (13).

Bohgaki et al. (20) evaluated IFN-γ and IL-4 production in T cells of SSc patients by intracellular staining. At inclusion, the cytokine production profile between transplanted SSc patients and healthy individuals was not significantly different (28). Frequencies of CD3^+^CD8^−^ and CD3^+^CD8^+^ cytokine-producing T cells were not different between poor or good response groups. Notably, IFN-γ producing CD8^+^ T cells increased after transplantation in both groups (28).

Tsukamoto et al. (21) showed that while AHSCT was effective in controlling disease activity of systemic sclerosis (SSc) patients, Th1/Th2 ratio was significantly increased for at least 3 years after transplantation. The authors showed that the IFN-γ^+^/ CD4^+^ T cells to IL-4^+^/CD4^+^ T cell ratio increased early after transplantation, reaching a plateau 6 months after AHSCT (29).

In addition, Crohn's disease patients who fully responded to treatment with AHSCT had higher numbers of regulatory T cells (Treg) and lower IFN- γ and IL-12 serum levels than non-responders (37). In juvenile arthritis patients, de Kleer et al. (31) demonstrated increased expression of mRNA IL-10 and decreased expression of mRNA IFN-γ in hsp60-specific T cells after transplantation (38). Therefore, authors suggest that AHSCT promotes a shift in autoreactive cells from pro-inflammatory to a more tolerant phenotype (38).

Type 1 diabetes (T1D) patients showed decreased levels of serum autoantibodies and of pro-inflammatory cytokines IL-1, IL-17, and TNF-α after AHSCT (24). Enders et al. (37) reported that patients with active juvenile dermatomyositis had elevated levels of three pro-inflammatory biomarkers (CXCL10, TNFR2, and Galectin-9) that highly correlated with disease activity. Notably, levels of these biomarkers decreased in two patients after transplantation (43).

In summary, the above-described reports showed interesting modulation of cytokine/chemokine/other soluble markers (13, 28, 29, 38, 43, 46, 50) after AHSCT. However, these post-transplantation changes only make sense if related to disease pathogenesis and/or activity. In our opinion, serum evaluation of the main classical cytokines (TNF-α, IFN-γ, IL-2, IL-4, IL-6, IL-8, IL-17, IL-18, IL-10, TGF-β) is interesting, but not essential for most AD post-AHSCT follow-up (Table 2). In organ-specific AD, serum concentration of most general cytokines is low and heterogeneous. However, in systemic AD serum levels of important soluble factors (such as cytokines chemokines, growth factors, and surrogate disease-specific markers) may be evaluated at pre- and post-transplantation periods to search for predictive biomarkers and/or treatment-response biomarkers of AHSCT for systemic AD (Table 2).

#### Regulatory T cell expansion

Autoreactive T cells that escape negative selection in the thymus are found in the peripheral blood of healthy individuals (53). However, peripheral tolerance mechanisms control autoimmunity and prevent development of autoimmune disease (62, 63). T cell-intrinsic (anergy, clonal deletion, or immunological ignorance) and extrinsic mechanisms (mediated by suppressor/regulatory cells) are essential to peripheral tolerance. The CD4^+^CD25^+^Foxp3^+^ Treg, the main natural regulatory T cell subset, are very important for extrinsic control of peripheral tolerance, once they modulate autoreactive T and B cell responses.

Almeida et al. (64) demonstrated that in lymphopenic environments CD8^+^ T-cell subsets impact on each other during expansion (64). CD4^+^CD25^+^ Treg suppress and control repopulation of CD8^+^ T cells, leading to balanced repopulation of central-memory and effector-memory T cell subsets (64). Therefore, CD4^+^CD25^+^ Treg may have a major role in regulating the expansion of CD8^+^ T cell subsets during repopulation of the lymphopenic environment after AHSCT in AD patients (55, 64).

In the context of AHSCT, de Kleer et al. (31) were first to demonstrate that AHSCT for AD induces immunologic self-tolerance by restoring the CD4^+^CD25^hi^ immune regulatory network (Table 1). AHSCT was able to normalize the frequency of CD4^+^CD25^hi^ Treg in patients with idiopathic juvenile arthritis (IJA). Recovery of normal Treg levels after transplantation was due to both, homeostatic expansion during the lymphopenic phase of immune reconstitution and thymic generation of naive Treg CD4^+^CD25^hi^ expressing FOXP3 mRNA (38). Since then, many groups have shown increased Treg numbers as an important and common immune mechanism of AHSCT for AD (16, 20, 23, 26, 31–33, 36–38, 41, 63) (Table 1). Delemarre et al. (34) demonstrated that remission of AD after transplantation involves renewal of the Treg TCR repertoire during thymic reactivation (41).

During immune reconstitution, the lack of Foxp3^+^ Treg results in development of “gaps” in the TCR repertoire and inappropriate responses to foreign antigens. Conversely, the presence of Foxp3^+^ Treg optimizes TCR repertoire diversity and foreign antigen responsiveness (64, 65). These studies provide an example of Treg activity that actually enhances immunity, which is very important in transplanted AD patients, contrasting with their generally accepted immunosuppressive function (64, 65).

The peripheral expansion of CD4^+^CD25^hi^Foxp3^+^ Treg and other regulatory populations during the lymphopenic phase post-transplantation is a very intriguing mechanism of AHSCT for AD. During this early phase of immune reconstitution, the total number of CD4^+^ T cells is very low. Therefore, expanded CD4^+^CD25^hi^Foxp3^+^ Treg cells correspond to the majority of CD4^+^ T cells in this phase. The expansion of cells with regulatory phenotype and function may allow the achievement of a “fine immune balance” in the patient until new naive T cells are produced by the reactivated thymus. Therefore, all future immune monitoring studies should assess the absolute number of phenotypically well-characterized Treg subsets and their immunosuppressive potential in well-designed assays (Tables 2, 3).

**Table 3:** Immunophenotyping of peripheral blood cell subsets in patients with AD undergoing AHSCT.

**CELL SUBSET**	**PHENOTYPE**
**Minimum panel for immune reconstitution analyses**	
Total CD3, CD4, or CD8 T cells	CD3^+^CD4^+^CD8^−^; CD3^+^CD4^−^CD8^+^
Recent thymic emigrants	CD3^+^CD4^+^CD31^+^CD45RA^+^CD45RO^−^
Naive T cells^**a**^, ^**b**^	CD3^+^CD4^+^(CD8^+^)CD45RA^+^CD45RO^−^CCR7^+^CD62L^+^
Central memory T cells^**a**^, ^**b**^	CD3^+^CD4^+^(CD8^+^)CD45RA^−^CD45RO^+^CCR7^+^CD62L^+^
Effector memory T cells^**a**^, ^**b**^	CD3^+^CD4^+^(CD8^+^)CD45RA^−^CD45RO^+^CCR7^−^CD62L^−^
Effector T cells^**a**^, ^**b**^	CD3^+^CD4^+^(CD8^+^)CD45RA^+^CD45RO^−^CCR7^−^CD62L^−^
Exausted T cells^**c**^	CD3^+^CD4^+^CD8^−^PD-1^+^; CD3^+^CD4^−^CD8^+^PD-1^+^
Naive B cells	CD19^+^CD27^−^IgD^+^
Switched memory B cells	CD19^+^CD27^+^IgD^−^
Non-Switched memory B cells	CD19^+^CD27^+^IgD^+^
Plasma cells	CD19^+^CD27^high^IgD^−^CD38^high^
Regulatory B cells (Breg)	CD19^+^CD24^high^CD38^high^ or CD19^+^CD24^high^CD38^high^IgM^high^IgM^high^CD5^+^CD10^+^CD20^+^CD27^−^CD1d^high^
Regulatory T cells (nTreg)	CD4^+^CD25^high^CD127^−^Foxp3^+^
Suppressor T cells (CD8)	CD8^+^CD28^−^CD57^+^PD1^+^
NK cells (cytotoxic)	CD3^−^ CD56^dim^CD16^+^
NK cells (cytotoxic)	CD3^−^ CD56^bright^CD16^+/−^
iNKT cells	CD19^−^CD3^+^Vα24^−^Jα18 TCR^+^
Stem cell-like memory T cells (TSCM)	CD3^−^CD4^+^(CD8^+^)CD45RO^−^CD45RA^+^CCR7^+^CD27^+^CD28^+^CD95^+^CD122^+^
**Other cell subsets for immune reconstitution analyses**	
Follicular T helper (Tfh)	CD3^+^CD4^+^CXCR5^+^PD-1^+^Bcl-6^+^FoxP3^−^ or CD3^+^CD4^+^CXCR5^+^PD-1^+^ICOS^+^
Th1 cells	CD3^+^CD4^+^CXCR3^+^CCR6^−^Tbet^+^ or CD3^+^CD4^+^CXCR5^−^CXCR3^+^
Th2 cells	CD3^+^CD4^+^CCR4^+^CCR6^−^GATA3^+^ or CD3^+^CD4^+^CXCR5^−^CCR4^+^CCR6^−^
Th17 cells	CD3^+^CD4^+^CCR6^+^CCR4^+^RORγt^+^ or CD3^+^CD4^+^CXCR5^−^CCR4^+^CCR6^+^CCR10^−^
Th22 cells	CD3^+^CD4^+^CCR10^+^CCR4^+^AHR^+^ or CD3^+^CD4^+^CXCR5^−^CCR4^+^CCR6^+^CCR10^+^
Monocytes (classic)	CD14^+^CD16^−^
Monocytes (inflammatory)	CD14^+^CD16^+^CD64^high^ CD32^low^
Monocytes (patrolling)	CD14^low^CD16^+^
Myeloid-derived suppressor cells (MDSCs) granulocytic^**d**^	Lin-CD14^−^HLADR^−^CD33^+^CD11b^+^
Myeloid-derived suppressor cells (MDSCs) monocytic^**d**^	Lin-CD14^+^HLADR^−^CD11b^+^
Plasmocytoid dendritic cells^**d**^	Lin^−^CD14^−^CD123^+^CD11c^−^
Conventional dendritic cells^**d**^	Lin^−^CD14^−^CD123^−^CD11c^+^
**Upon** ***in vitro*** **t or b cell activation**	
Signaling Pathways (Erk, p38MAPK)^e^	CD3^+^CD4^+^CD8^−^ERK; CD3^+^CD4^+^CD8^−^p38MAPK
Cytokine-producing CD4^+^ ou CD8^+^ T cells	CD3^+^CD4^+^(CD8^+^) IL2^−/+^IL4^−/+^IL17^−/+^TNFα^−/+^IL10^−/+^IFNγ^−/+^
IL-10-producing Breg cells	CD19^+^CD24^high^CD38^high^IL10^+^
IL-10-producing Treg cells	CD4^+^CD25^high^CD127^−^Foxp3^+^IL10^+^

a *CD4^+^(CD8^+^) = CD4^+^ or CD8^+^ T cells*.

b *CD45RO and CD62L are not essential to the panel*.

c *Other exhaustion markers (such as TIM-3, LAG-3) or activation markers (CD69, HLA-DR) can be used*.

d *Lineage (Lin) cocktail: CD3/CD19/CD20/CD56. Immunophenotyping panels were based on previous reports (12, 66, 67, 68, 69)*.

#### Regulatory B cell expansion

Regulatory B cells (Breg) are immunosuppressive cells that support immunological tolerance (70, 71). They modulate the immune responses mainly via IL-10 secretion (70, 71). Abnormalities in the Breg number or function have been described in many immune-mediated disorders. Therefore, Breg are considered essential to maintain immune homeostasis (70, 71). CD19^+^CD24^hi^CD38^hi^ B-cell populations are well-characterized Breg in humans, playing an important role in the control of autoreactivity (72).

Recently, our group (31) showed that Breg frequencies transiently increased after AHSCT, tending to remain higher than pre-transplant values for at least 2 years (Table 1). We believe that Breg may be involved in the reestablishment of auto-tolerance after AHSCT, as suggested by persistently increased Breg/memory B cell ratio, as well as higher IL-10 production in SSc patients after transplantation (31). Notably, we found that Breg expansion occurs early post-transplantation [(31) and unpublished observation of ongoing studies]. Therefore, peripheral blood samples should be collected at 1, 3, and 6 months after transplantation (Tables 2, 3). In our opinion, whenever possible, besides the classical CD19^+^CD24^hi^CD38^hi^ Breg, other regulatory B cell subsets (71) should be evaluated. In addition, functional assays to evaluate the immunosuppressive capacity of reconstituted Breg populations are also desirable (72).

#### Homeostatic proliferation and CD8^+^CD28^−^CD57^−^ expansion

After transplant-induced immunological depletion, lymphocytes repopulate the immune space both through enhanced T cell neogenesis from the thymus and proliferation of residual non-depleted peripheral lymphocytes, in a biological process named homeostatic proliferation. At this condition, CD8^+^ T cells proliferate more than the CD4^+^ T cell population (73).

Abrahamsson et al. (39) showed that CD8^+^CD28^−^CD57^+^ T cell subsets were persistently increased in MS patients after AHSCT and were able to suppress CD4^+^ T cell proliferation with variable potency (17). CD8^+^CD28^−^CD57^+^ T cells have regulatory properties and their numbers are usually found decreased in AD (74) (Table 1). In this context, we suggest that CD8^+^CD28^−^CD57^+^ T regulatory T cells expand preferentially following peripheral immune homeostasis disruption, such as in the early post-AHSCT period. In addition, many other mechanistic studies have also demonstrated that this regulatory/suppressor T cell subset expands after transplantation, indicating its important role in AHSCT for AD (Table 1).

We have also recently found in SSc patients that homeostatic proliferation after AHSCT results in transient telomere attrition and increased numbers of senescent and exhausted CD8^+^CD28^−^CD57^+^ T cells. In addition, high expression of programmed cell death protein 1 (PD-1) in peripheral T cells is associated with better clinical outcomes after AHSCT (48).

In our opinion, this T cell population plays and important role in controlling autoimmunity early post-AHSCT. Future immune monitoring studies should better characterize their phenotype, their immunosuppressive capacity and possibly other mechanisms of action in AD patients undergoing AHSCT (Tables 2, 3). Our studies have suggested that these CD8^+^CD28^−^CD57^+^ T cells express PD-1 (73), and perhaps other immunoregulatory molecules, as supported by recent literature from other researcher groups (75).

#### Increased PD-1 expression on T and B cells

During T cell activation, the PD1 (or CD279) molecule is expressed on the cell surface and may engage its ligand, the programmed cell death ligand 1 (PDL1; also known as CD274) and/or PDL2 (also known as CD273). Upon binding, positive signals are generated through TCR and CD28 (76). These co-inhibitory receptors act as immune checkpoints for effector T cells, which regulate the adaptive immune responses. In fact, mice deficient in PD-1 molecules are susceptible to development of autoimmunity and AD, as reviewed by Sharpe and Pauken (77). Moreover, PD-1 polymorphisms detected in human autoimmune disorders support a role for the PD-1 pathway in self-tolerance mechanisms (77). Of note, during viral infections, the PD-1 molecule is upregulated on the surface of T cells during acute homeostatic proliferation (78, 79).

Thangavelu et al. (80) showed that recent thymic emigrants deficient in PD-1, which were generated after transfer of PD-1 deficient hematopoietic stem cells into lymphopenic adult Rag-deficient mice, induced a systemic inflammatory disease (80). Therefore, under lymphopenic conditions, PD-1 signaling is essential for systemic self-tolerance. In addition, Ellestad et al. (81) demonstrated that the most important role of PD-1 pathway is not to promote Treg expansion, but to control T cell activation and proliferation in response to self-antigens (81). Therefore, in lymphopenic environments, the PD-1 pathway is essential to regulate T cell activation and proliferation and consequently to avoid development of autoimmunity and AD.

In this context, Arruda et al. (43) showed that MS patients treated with AHSCT had expansion of PD-1^+^CD19^+^ B-cells and PD-1^+^CD8^+^ T-cells early post-transplantation. Therefore, the PD-1 inhibitory pathway is considered an immune regulatory mechanism by which AHSCT restores auto-tolerance in patients with MS (45) and other AD (Table 1).

Our group has recently shown increased expression of PD-1 in T cells from SSc patients who responded to AHSCT. As a general mechanism to keep potentially autoreactive CD8^+^ T-cell clones under control after AHSCT, PD-1 expression may be a reliable immune marker of clinical response in SSc and MS patients after AHSCT (48) (Table 1). Based on these recent data, we recommend the evaluation of expression of PD1 on T and B cell subsets in future immune monitoring studies (Tables 2, 3). In addition, we suggest to evaluate the expression of Tim-3 (T cell immunoglobulin-3), Lag-3 (Lymphocyte activation gene-3 or CD223) and TIGIT (T cell immunoglobulin and ITIM domain), other co-inhibitory receptors with specialized functions in immune regulation, on T cells (82).

#### Decreased autoreactivity early Post-AHSCT

Elimination of autoreactive effector memory T cells in patients with AD is supposed to ameliorate the autoimmune aggression and diminish disease activity. Sun and colleagues showed reduced T cell responses to myelin basic protein in the reconstituted immune system of MS patients after AHSCT, when compared to pre-transplant levels (13) (Table 1).

Recently, we showed that pre-transplant frequencies of autoreactive CD8^+^ T cells in T1D patients predicted the duration of insulin independency after AHSCT (26). Additionally, T1D patients that remained insulin free for longer periods also had persistently lower frequencies of autoreactive CD8^+^ T cells compared with patients who resumed insulin after transplantation (26) (Table 1).

In our opinion, monitoring of antigen-specific autoreactivity should be performed routinely in immune monitoring studies, as reported previously (13, 17, 26). We acknowledge the enormous difficulties to quantify peripheral autoreactive CD4^+^ or CD8^+^ T cells based on the variety of autoantigens/peptides and class-I or class-II HLA molecules. However, collaborations should be settled with autoimmunity expert groups for monitoring autoreactive T cells on frozen PBMC patient samples or researchers could use commercially available HLA-tetramers.

We believe it would be very important to also investigate if late reactivation of the AD after AHSCT coincides with the increase of new autoreactive naive T cells produced by the reactivated thymus or/and with a decrease in immune regulatory networks (Treg and Breg cell numbers and/or function), triggered by environmental factors, leading to loss of the once achieved “fine immune balance.”

Current data indicate that more intense immunoablation is associated with more favorable clinical outcomes after AHSCT (26, 31, 32, 35, 45) due to greater depletion of autoreactive T and B cells. However, complete eradication of the existing autoreactive immunologic memory may not be possible, even under high-intensity myeloablative conditioning regimens, since memory T and B cells reside in the bone marrow, are present in other body tissues and may survive immunoablative regimens (51, 83).

Currently, it is not known if immunosuppressive agents can infiltrate the tissues beyond the bone marrow. In experimental models, it has been shown that T cell depletion by ATG is more efficient in the blood compared with peripheral lymphoid organs (84). Park and Kupper (83) have demonstrated that tissue resident memory T cells are not totally depleted in non-barrier tissues after high dose immunosuppressive therapy (83). Curiously, in patients with refractory Crohn's disease, it was recently shown that the TCR repertoire diversifies after AHSCT in their intestinal tissue. The authors showed significant resetting of the TCR repertoire, since only 20% of TCR sequences were detected pre-transplantation and also 6 and 12 months post-AHSCT periods (49), prior to the production of any new naive T cells.

## Disease-specific immune mechanisms after AHSCT for AD

So far, few disease-specific immune mechanisms of AHSCT have been reported. These mechanisms include quantification of disease-specific autoreactive T cells and their characterization (cytokine-profile, mRNA expression) (13, 16, 38); identification/quantification/functional characterization of different regulatory T cell subsets (33); and quantification of cytokine and other soluble markers related to disease pathogenesis (16, 17).

Darlington et al. (36) demonstrated that myelin-specific T cells reconstitute after AHSCT in all transplanted MS patients. These autoreactive T cells may expand from residual cells or may be newly generated by the thymus. Re-emerged myelin-specific T cells had the same Th1 and Th2 baseline profile, but Th17 cells decreased after transplantation, as demonstrated by lower RORγ expression and IL-17A serum concentration. In addition, levels of IL-1β and IL-6 were also decreased in MS patients after AHSCT (16). In MS patients, Muraro et al. (14) showed increased expression of Fas on CD4^+^ and CD8^+^ T cells after AHSCT (14). Moreover, the same group has shown that the pro-inflammatory CD8^+^CD161^high^ mucosal-associated invariant T cell (MAIT) subset was depleted from the peripheral blood of MS patients after AHSCT (17) (Table 1).

Most reported studies have evaluated classical regulatory T cell subsets (Table 1) in the context of AHSCT for AD, but Zhang et al. (26) identified peripheral CD8^+^ T cells from SLE patients with sustained high FoxP3 expression and increased expression of CTLA-4, PD-1, PD-L1, latency-associated peptide (LAP), and CD103, when compared with pre-transplant CD8^+^ T cells. The CD8^+^ Treg subset reconstituted post-transplantation presented increased suppressive activity, both autoantigen-specific and non-specific, which was predominantly TGF-β dependent and contact-independent. Therefore, the generation of a new LAP^high^CD103^high^CD8^+^ Treg subset was reported, improving the immune regulatory deficiency and correlating with clinical remission (33) (Table 1).

In summary, we cannot conclude that the immune mechanisms cited above are disease-specific because they have not been yet evaluated in all AD subtypes currently treated with AHSCT. For future immune monitoring studies, we suggest the evaluation of validated disease-specific biomarkers (closely related to disease pathogenesis) in patients with AD undergoing AHSCT therapy (85–93).

## Establishing biomarkers of AHSCT for autoimmune diseases

Recent mechanistic studies of AHSCT for AD are more complete and comprehensive. Higher numbers of transplanted patients, longer follow-up, different methodologies, and modern experimental approaches have contributed to the understanding of the reconstituting immune system. This mechanistic knowledge can be now reverted to the clinic, improving transplantation protocols and clinical outcomes. Moreover, the identification of new biomarkers may further enhance transplantation efficacy.

Biomarkers are urgently needed to improve disease diagnosis, to monitor disease activity and therapeutic responses, and to allow validation of new therapies (94–96). A biomarker is defined as a biological substance that can be quantified and dynamically evaluated in the context of normal biological or pathogenic processes and their progression, or in the context of clinical responses to therapeutic interventions. Biomarkers must be reproducible, robust and validated (94–96).

Mechanistic biomarkers may inform and validate how a particular treatment works. Over the past years, many mechanistic biomarkers of AHSCT for AD have been described and validated, based on different clinical outcomes (response and lack of response to treatment). In this context, increased PD-1 expression and expansion of Treg and Breg cells associate with a favorable clinical response to AHSCT in SSc, MS, and T1D patients (Table 1, Figure 1).

In addition, predictive biomarkers are disease-associated and indicate whether there is a chance of disease activation. They can also measure, at time of enrollment, the expected patient responsiveness to a specific treatment. Karnell et al. (15) proposed the increased numbers of memory CD4^+^ and CD8^+^ T cells at pre-transplantation as predictive biomarkers for response in MS patients. In addition, cumulative frequency of autoreactive islet-specific CD8^+^ T cells at pre-transplantation predicts the clinical outcome of AHSCT in T1D patients (26). After AHSCT, T1D patients with higher autoreactivity before transplantation reactivated disease earlier than patients with lower autoreactivity (26).

Biomarkers that predict the future outcome of a patient with a specific disease, and also the overall survival rates and clinical benefits from a therapeutic intervention, are called prognostic biomarkers. Several clinical studies demonstrated that remission of AD after AHSCT associates with increased TCR repertoire diversity due to thymic reactivation and to the decrease of effector and memory T cell numbers (14, 27, 32, 39, 40). In this context, thymic reactivation may be considered a prognostic biomarker, associated with positive clinical outcomes (Table 1, Figure 1).

Additional prognostic biomarkers have been defined for AHSCT in AD, such as increased Treg frequencies and reduced inflammatory cytokine levels (IFN-γ and IL-12) in responsive Crohn's disease patients (37), increased frequencies of T and B cells expressing PD-1 in responsive MS patients (62), reduced levels of inflammatory markers (TNFR2, CXCL10, and GAL-9) in responsive juvenile dermatomyositis patients, and increased Treg numbers in long-term responsive T1D patients (26). More recently, Arruda et al. (48) demonstrated that clinical improvement of SSc patients is related to increased numbers of newly generated Treg and Breg after AHSCT, as a result of coordinated thymic and bone marrow rebounds (31) (Table 1, Figure 1).

## Conclusions and future directions

Since the publication of Sun et al. (13), we have increased our knowledge on mechanistic biomarkers of AHSCT (Table 1). It is now clear that most immune mechanisms are common to several AD and depend more on the conditioning regimen and quality of immune reconstitution, rather than only on disease pathogenesis (Table 1). Every parameter that affects the quality of immune reconstitution (e.g., infections, patient age, conditioning regimen, previous treatments) may affect clinical outcomes after AHSCT. Most patients respond to AHSCT and achieve long-term disease remission, however a subset of patients reactivate the AD after transplant (1, 4, 7–10) (Figure 1). Therefore, additional immune interventions are urgently warranted to improve AHSCT protocols.

In our opinion, we have reached another level of investigation in the field of AHSCT for AD. Future perspectives are protocol improvements (e.g., modifications in the conditioning regimens) and combined therapies (e.g., infusion of *in vitro* expanded immune regulatory cells, immune modulatory drugs). These may vary according to AD pathogenesis (Figure 1).

Strategies can be directed to improve specific immune regulatory mechanisms of AHSCT. For example, to increase the number and/or function of regulatory CD4^+^ or CD8^+^ T cell subsets in patients who did not respond sufficiently to the AHSCT as a single treatment, we could propose combined therapies with similar immune mechanisms of action, such as administration of intravenous immunoglobulin (IVIG) (97), vitamin D (98) or low-dose rapamycin (99), infusion of autologous expanded Treg (100), or infusion of autologous or allogeneic mesenchymal stromal cells (101).

The ultimate importance of immune monitoring evaluations in patients undergoing AHSCT for AD is their impact in the clinical setting and consequent contributions to improve safety and efficacy of clinical protocols. Here, we provide three examples of how immune monitoring evaluations have already impacted the AHSCT scenario.

In 2004, Sun et al. (13) showed that myelin-specific T cells were not detected in MS patients at early periods post-AHSCT but that they reconstituted 1 year post-transplantation. Similarly, our group has shown that type 1 diabetes patients with higher frequencies of autoreactive islet-specific T cells before transplantation reactivate the disease earlier than patients with lower frequencies of these cells (26). These data indicate that conditioning regimens might have different efficacy on different AD and/or patients depending on their immune status and/or level of autoreactivity before transplantation. Indeed, our study has shown that high dose immunosuppression was not strong enough to sufficiently deplete autoreactive islet-specific T cells (26). Based on these observations, our center has increased the immunoablative intensity of the conditioning regimen protocol from 200 mg/Kg of cyclophosphamide plus 4.5 mg/Kg of rabbit anti-thymocyte globulin (ATG), to 120 mg/Kg of cyclophosphamide plus 150 mg/Kg of fludarabine and 4.5 mg/Kg of ATG, to further increase T and B cell ablation.

In the second example, Dubinsky et al. (25) demonstrated that T-cell clones persisting in the peripheral blood after autologous hematopoietic SCT were undetectable in the CD34^+^ selected graft (16). We can therefore assume that the residual autoreactive cells, responsible for disease reactivation in some patients, did not emerge from the CD34^+^ autograft, but most probably survived the immunoablative regimen. In this context we recommend, for future mechanistic investigations, the evaluation of graft composition (all T and B naive and memory subsets, regulatory T and B cell subsets, autoreactive cells) after CD34^+^ cell mobilization for each transplanted AD disease, as well as its correlation with clinical outcomes.

Third, some studies have evaluated how CD34^+^ graft selection influences the immune reconstitution after AHSCT (1). Recently, Keever-Taylor et al. (102) showed that the manufacturing of autologous CD34^+^ cells in the “High-Dose Immunosuppression and Autologous Transplantation for Multiple Sclerosis” (HALT MS) and “Scleroderma: Cyclophosphamide or Transplantation” (SCOT) protocols was comparable across all transplantation centers and allowed successful granulocyte and platelet recoveries. On the other hand, Oliveira et al. (44) have demonstrated that CD34^+^ selection does not add benefit to the outcomes of transplanted SSc patients. These findings should be further confirmed by prospective randomized trials (22).

Immune monitoring and biobanking guidelines for AHSCT in patients with AD patients have been established by the European Blood and Marrow Transplantation (EBMT) group, in 2015 (12). They have provided very reliable recommendations for comparative research studies envisioning biomarker discovery (12). We believe EBMT guidelines for immune monitoring are still valid, especially for disease-specific evaluations. However, new data on the topic have been made available in the past 3 years, and we believe it is important to add them to this perspective, as additional/complementary recommendations (12) (Figure 1, Tables 2, 3).

Here, we encourage standardization of immune monitoring studies throughout transplantation centers worldwide associated with data registries of clinical and laboratory results, aiming to empower statistical analyses. Nowadays, with modern biobanking infrastructure and internationalization trends it should be easier to manage multicenter immune monitoring studies in order to identify potential and consistent biomarkers.

Discovery and development of biomarkers are definitely an unmet need in the field of AHSCT for AD. Some biomarkers can serve as early surrogates of eventual clinical outcomes or guide therapeutic decisions by enabling identification of individuals likely to not respond to this therapy (50, 85–87, 103).

In recent years, both traditional and next-generation applications, including large-scale transcriptomic, epigenomic, genomic, lipidomic, cytomic, and proteomic technologies have yielded a huge amount of new candidate biomarkers that correlate with different clinical phenotypes of AD. Researchers should now focus on discovering and developing such biomarkers that would allow the improvement of clinical protocols and therapeutic efficacy of AHSCT for AD (57–61).

We encourage the scientific community to discuss future therapeutic directions and to establish updated guidelines of immune monitoring studies in order to expand the field. The following points should be addressed in the future, to better understand and subsequently improve AHSCT-AD outcomes:

- Establish a standardized immune monitoring platform for AHSCT-AD clinical trials: (a) to harmonize immune reconstitution results allowing centers to gather data/results and perform meta-analysis; (b) to invest in biomarker discovery by modern technologies and appropriate biobanking logistics (see recommendations on Tables 2, 3, and Figure 1);
- Conduct additional multicenter clinical trials to harmonize clinical and also immune monitoring data, allowing significant, and conclusive results about AHSCT-AD efficacy and immune mechanisms;
- Establish standardized conditioning regimens for each AD, based on the recent experiences and clinical achievements from each group. Since conditioning regimens have great impact on immune reconstitution and potentially on clinical outcomes, there is an urgent need for standardization. Later, more personalized conditioning approaches may be allowed, based on the AD disease and patient immune status.
- Develop combined therapies to improve therapeutic efficacy of AHSCT in AD patients who do not respond sufficiently to transplantation as a single treatment (Figure 1).

## Ethics statement

This study has been approved by the Brazilian institutional review board, where the patients were enrolled, and complied with country-specific regulations. The study was conducted according to the Declaration of Helsinki and Good Practice Guidelines. All patients read and signed informed consents.

## Author contributions

KM and MO wrote the manuscript. LA, JL-J, JdA, GdO performed literature review data analysis. All authors revised the manuscript and contributed to the final editing. All authors agreed with the content of the manuscript.

### Conflict of interest statement

The authors declare that the research was conducted in the absence of any commercial or financial relationships that could be construed as a potential conflict of interest.
